# Supplementary material for: Bioguided Isolation of Alkaloids and Pharmacological Effects of the Total Alkaloid Fraction from Aspidosperma pyrifolium Mart. (Apocynaceae)
Source: Plants (Basel). 2021 Nov 20;10(11):2526. doi: 10.3390/plants10112526 (PMC8625095; doi:10.3390/plants10112526)
Supplement: Supplementary file 1 [file plants-10-02526-s001.zip › plants-1468555-supplementary.pdf]

Supplementary Material

Bioguided isolation of alkaloids and pharmacological effects of the total alkaloid fraction from *Aspidosperma pyrifolium* Mart. (APOCYNACEAE)

Content

General Experimental Procedures
Figure S1. UV spectrum of **1** and **2**
Figure S2. ^{1}H NMR spectrum of **1** and **2** (500 MHz, acetone-d_6)
Figure S3. ^{1}H NMR spectrum expansion of **1** and **2** (500 MHz, acetone-d_6)
Figure S4. ^{1}H NMR spectrum expansion of **1** and **2** (500 MHz, acetone-d_6)
Figure S5. ^{1}H NMR spectrum expansion of **1** and **2** (500 MHz, acetone-d_6)
Figure S6. ^{13}C NMR spectrum of **1** and **2** (125 MHz, acetone-d_6)
Figure S7. ^{13}C NMR spectrum expansion of **1** and **2** (125 MHz, acetone-d_6)
Figure S8. HSQC spectrum of **1** and **2** (acetone-d_6)
Figure S9. HSQC spectrum expansion of **1** and **2** (acetone-d_6)
Figure S10. HMBC spectrum of **1** and **2** (acetone-d_6)
Figure S11. HMBC spectrum expansion of **1** and **2** (acetone-d_6)
Figure S12. HMBC spectrum expansion of **1** and **2** (acetone-d_6)
Figure S13. NOESY spectrum expansion of **1** and **2** (acetone-d_6)

General Experimental Procedures

The fraction was diluted in 0.2% aqueous trifluoroacetic acid (TFA):acetonitrile (ACN) solution (1:1 – v:v) to 1 mg mL^{-1} and the volume injected into the device was 50 μL. Reverse phase column C-18 maintained at 40° C and, as mobile phase, 0.2% TFA:ACN aqueous solution (77:23 to 65:35 v: v) was used with 10 mL flow rate. min^{-1}, 15 minutes, gradient mode. The sample was injected on preparative HPLC (2767 Sample Manager system, Waters, with UV-Vis photodiode detector). ^{1}H and ^{13}C NMR spectra (500 and 125 MHz, acetone-d_6) (1D and 2D) were recorded on a Varian Mercury spectrometer using TMS as the internal standard.

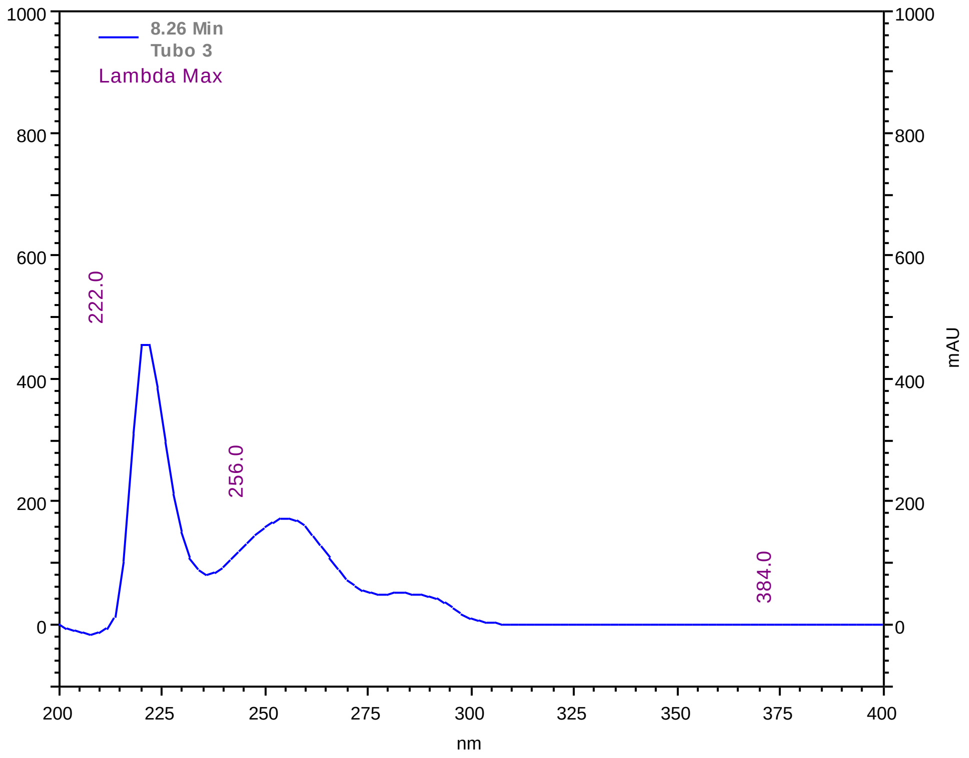

Figure S1. UV spectrum de **1** and **2**

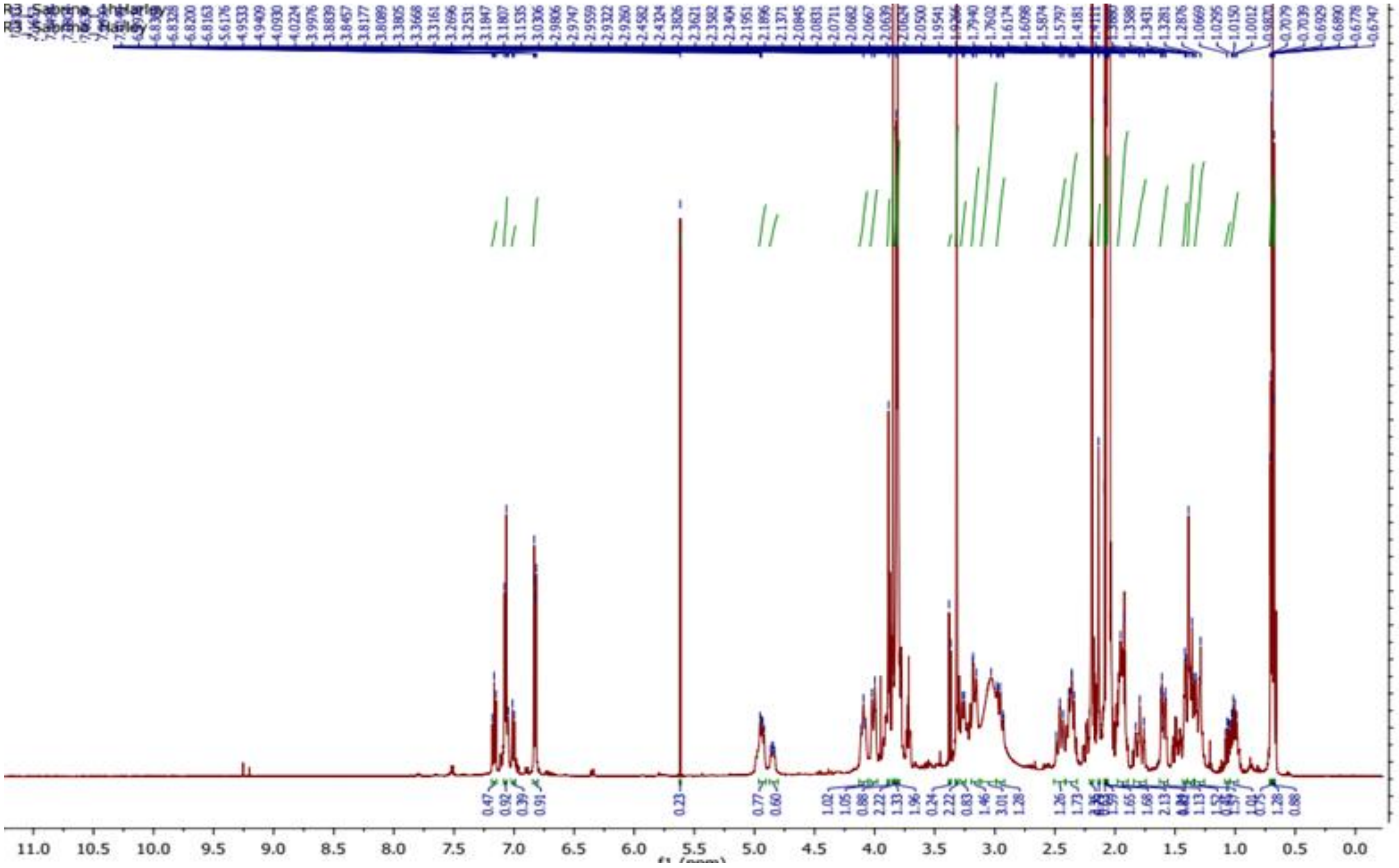

Figure S2. ^{1}H NMR spectrum of **1** and **2** (500 MHz, acetone-d_6)

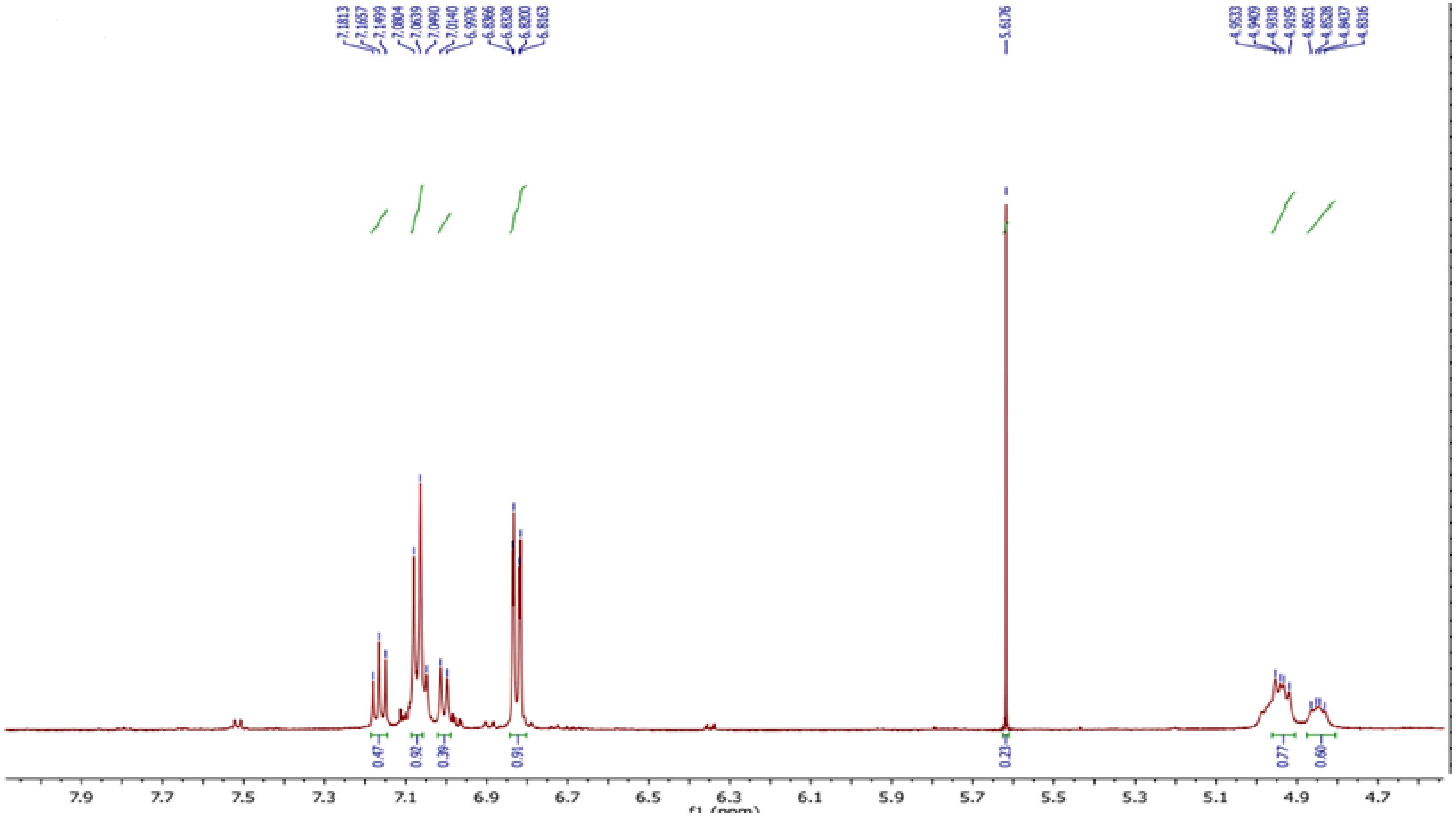

Figure S3. ^{1}H NMR spectrum expansion of **1** and **2** (500 MHz, acetone-d_6)

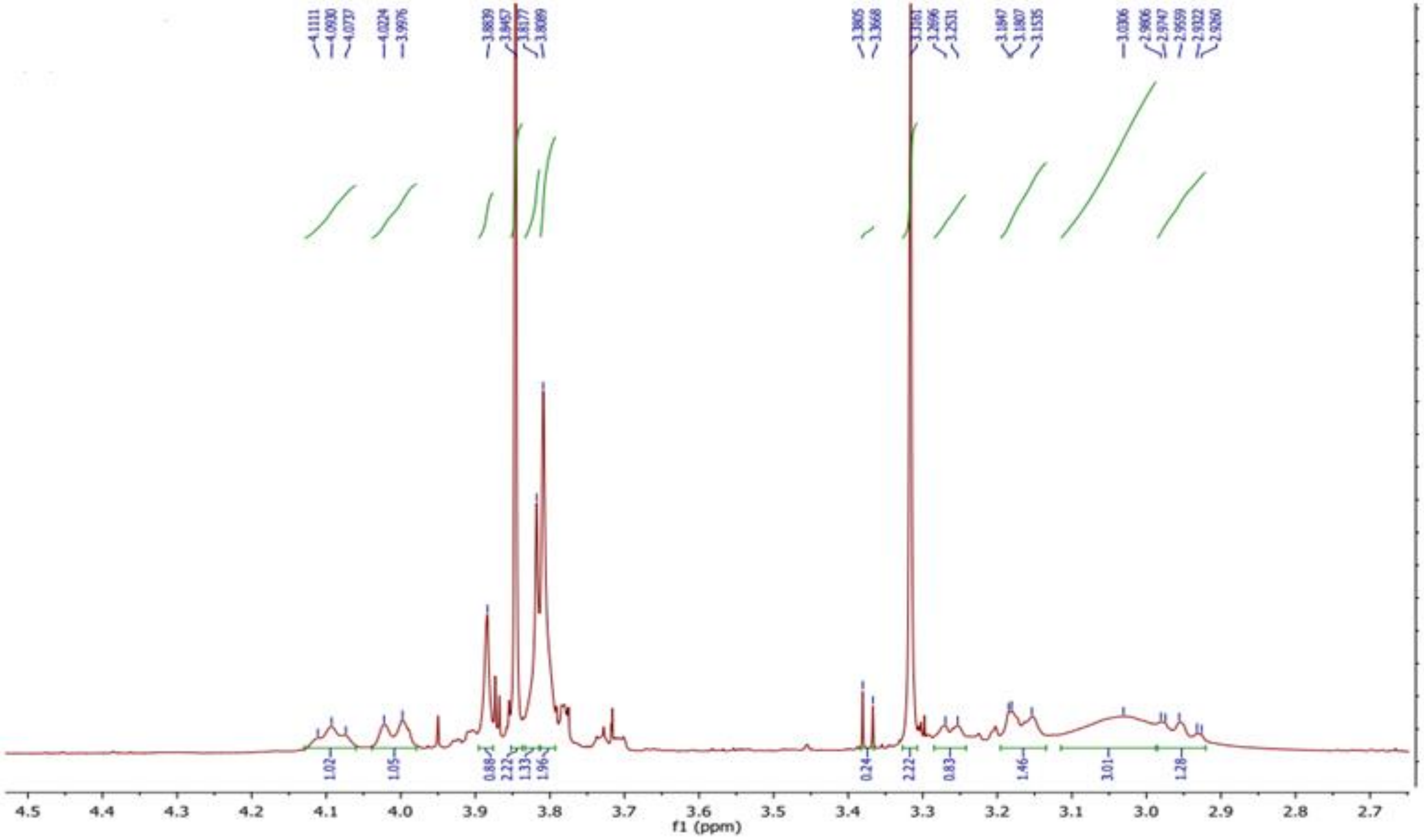

Figure S4. ^{1}H NMR spectrum expansion of **1** and **2** (500 MHz, acetone-d_6)

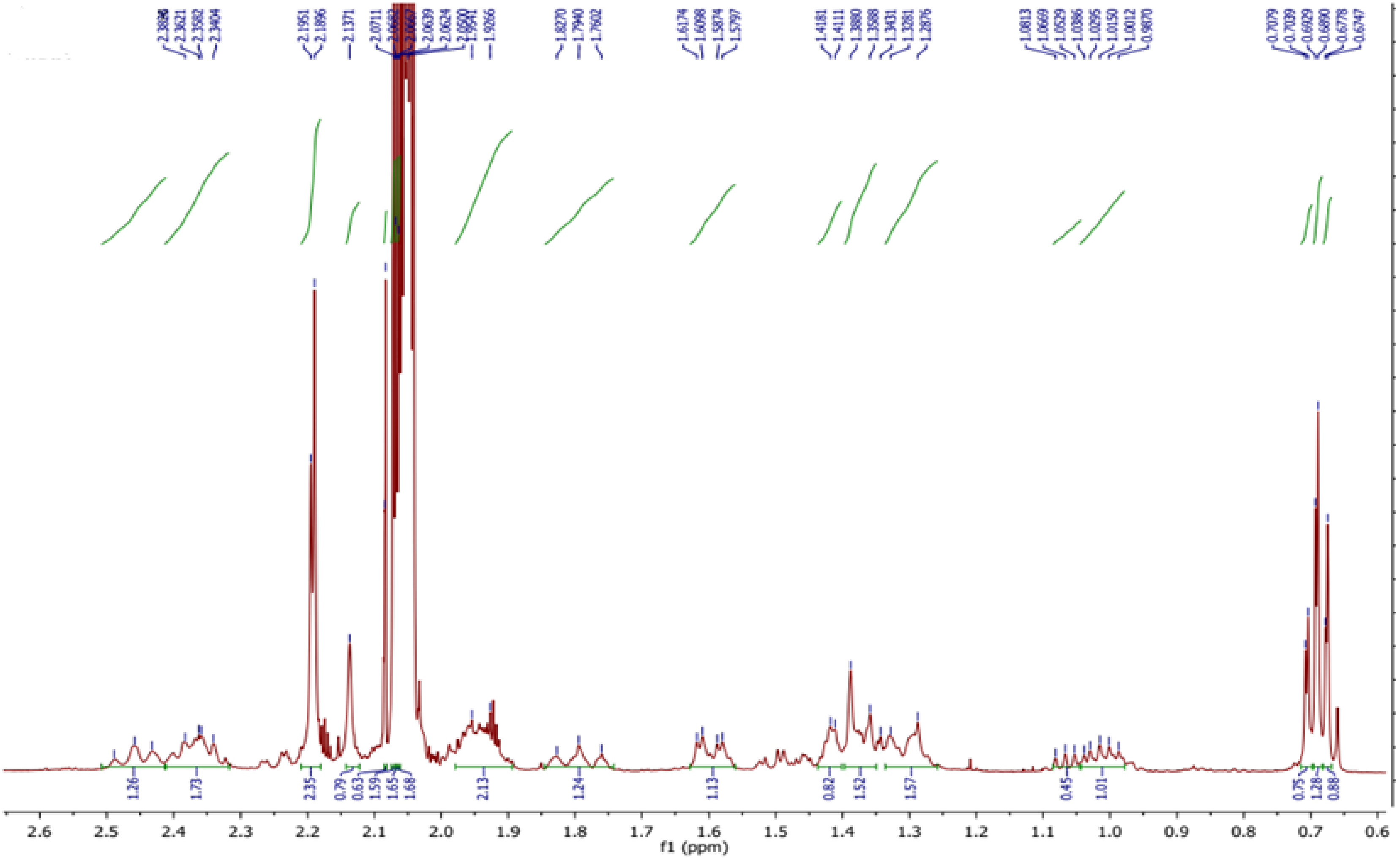

Figure S5. ^{1}H NMR spectrum expansion of **1** and **2** (500 MHz, acetone-d_6)

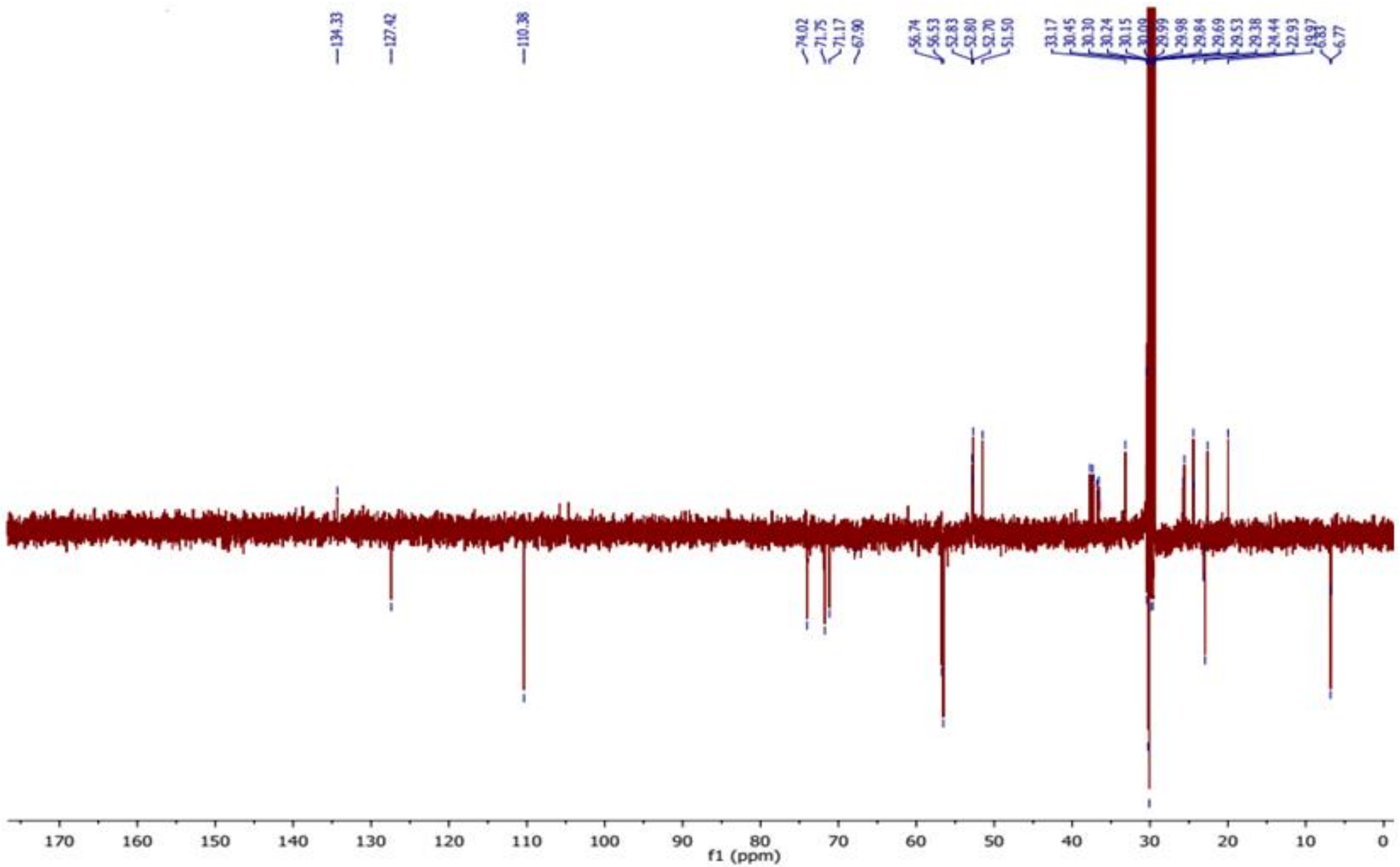

Figure S6. ^{13}C NMR spectrum of **1** and **2** (125 MHz, acetone-d_6)

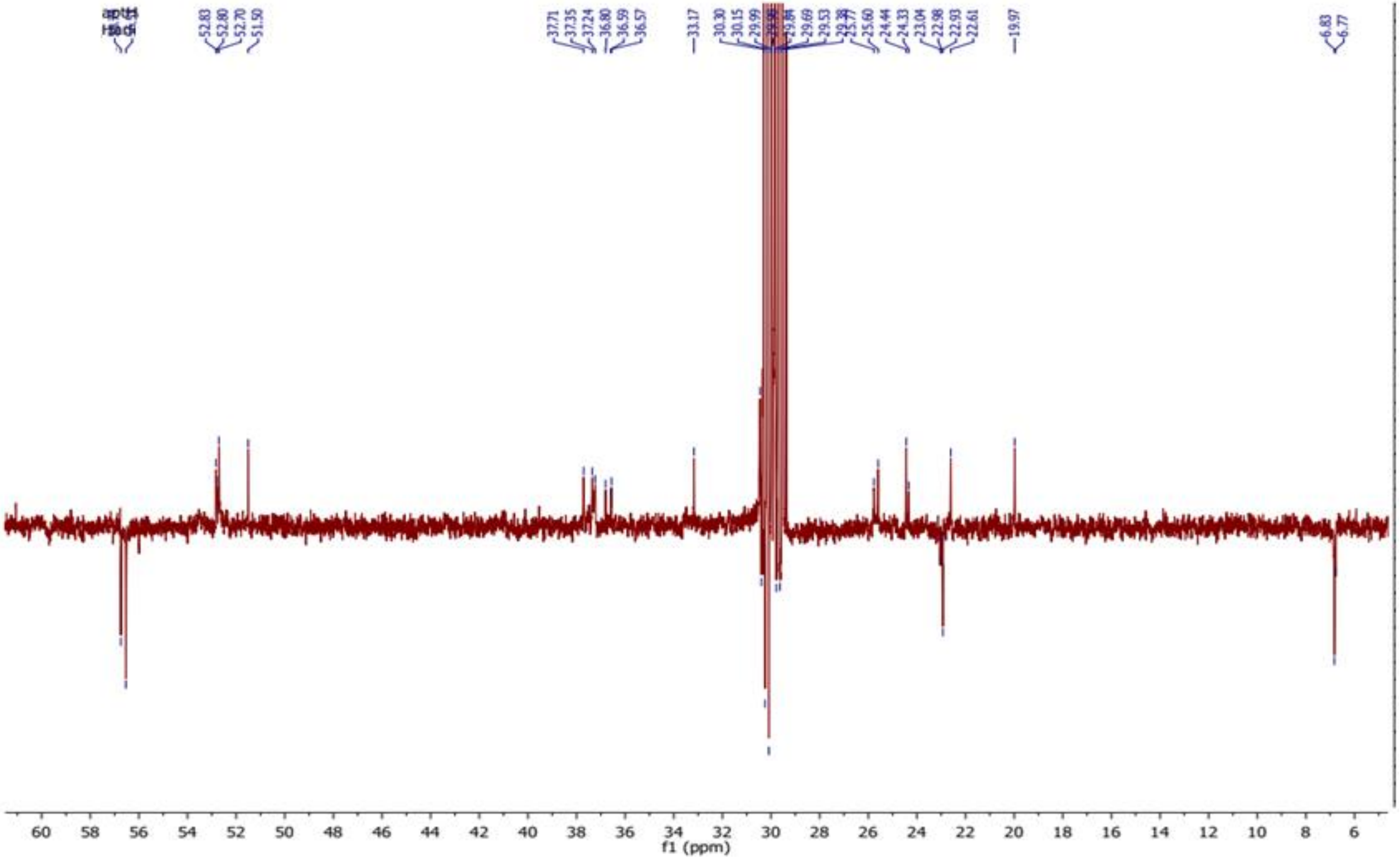

Figure S7. ^{13}C NMR spectrum expansion of **1** and **2** (125 MHz, acetone-d_6)

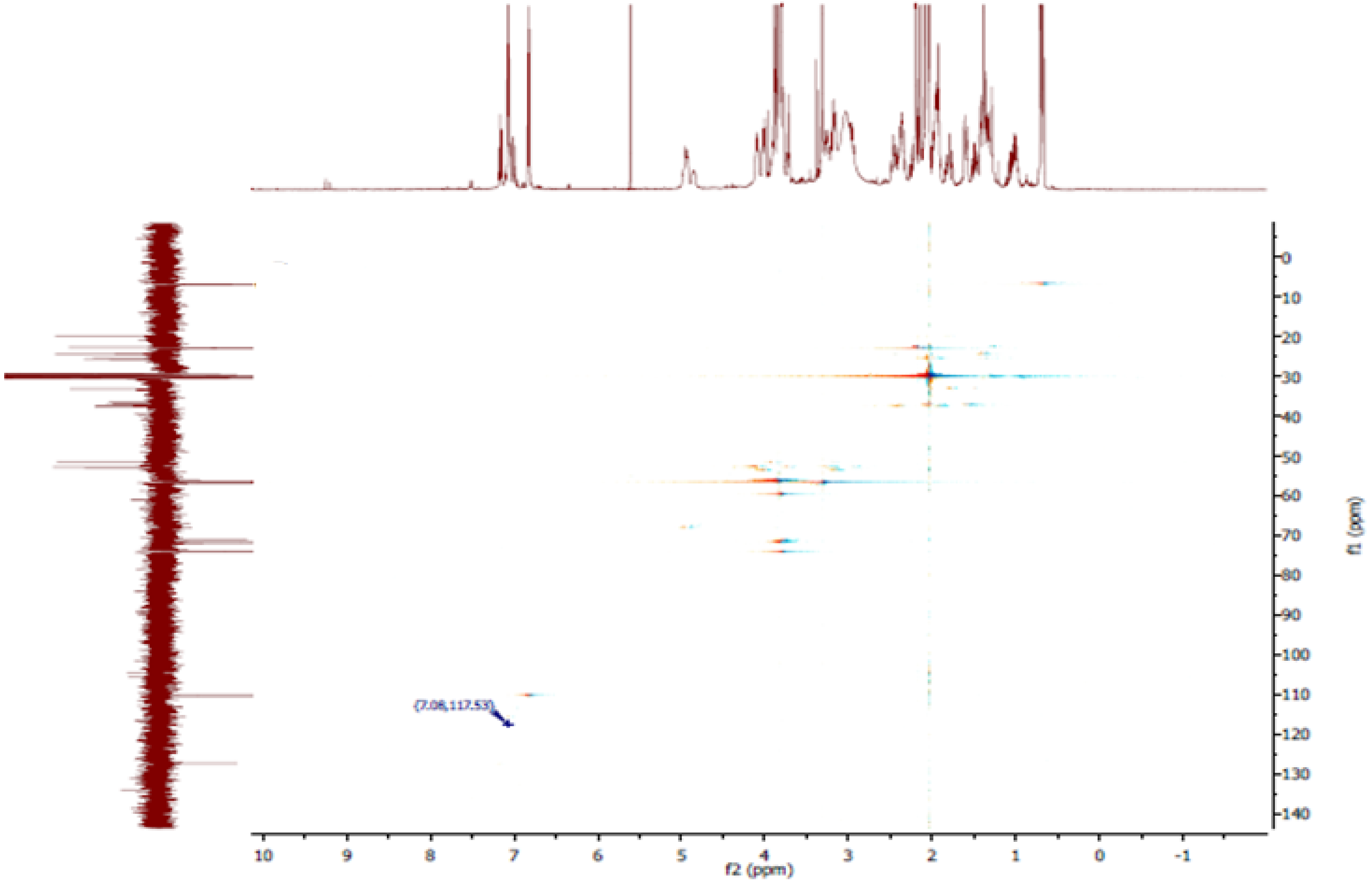

Figure S8. HSQC spectrum of **1** and **2** (acetone-d_6)

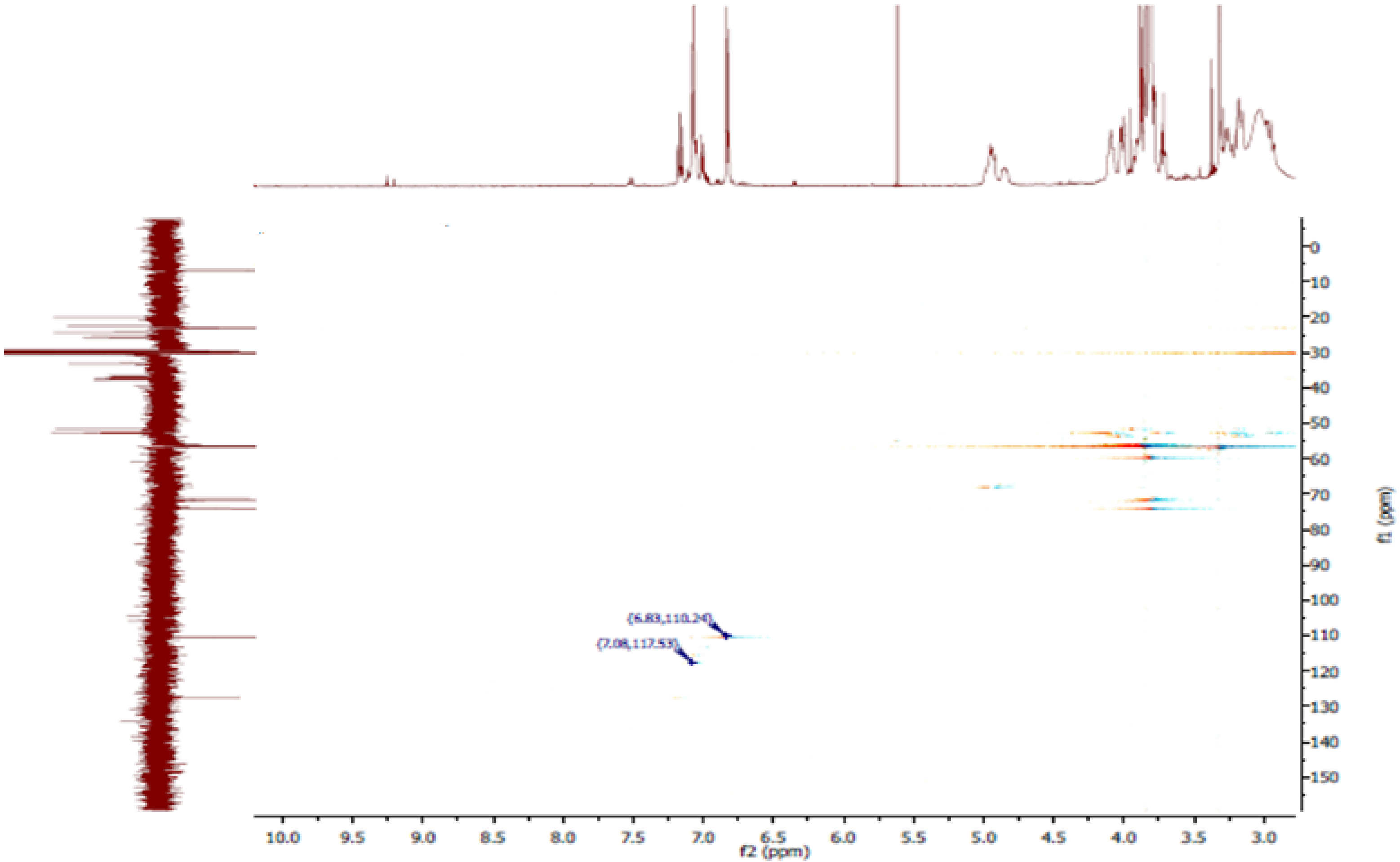

Figure S9. HSQC spectrum expansion of **1** and **2** (acetone-d_6)

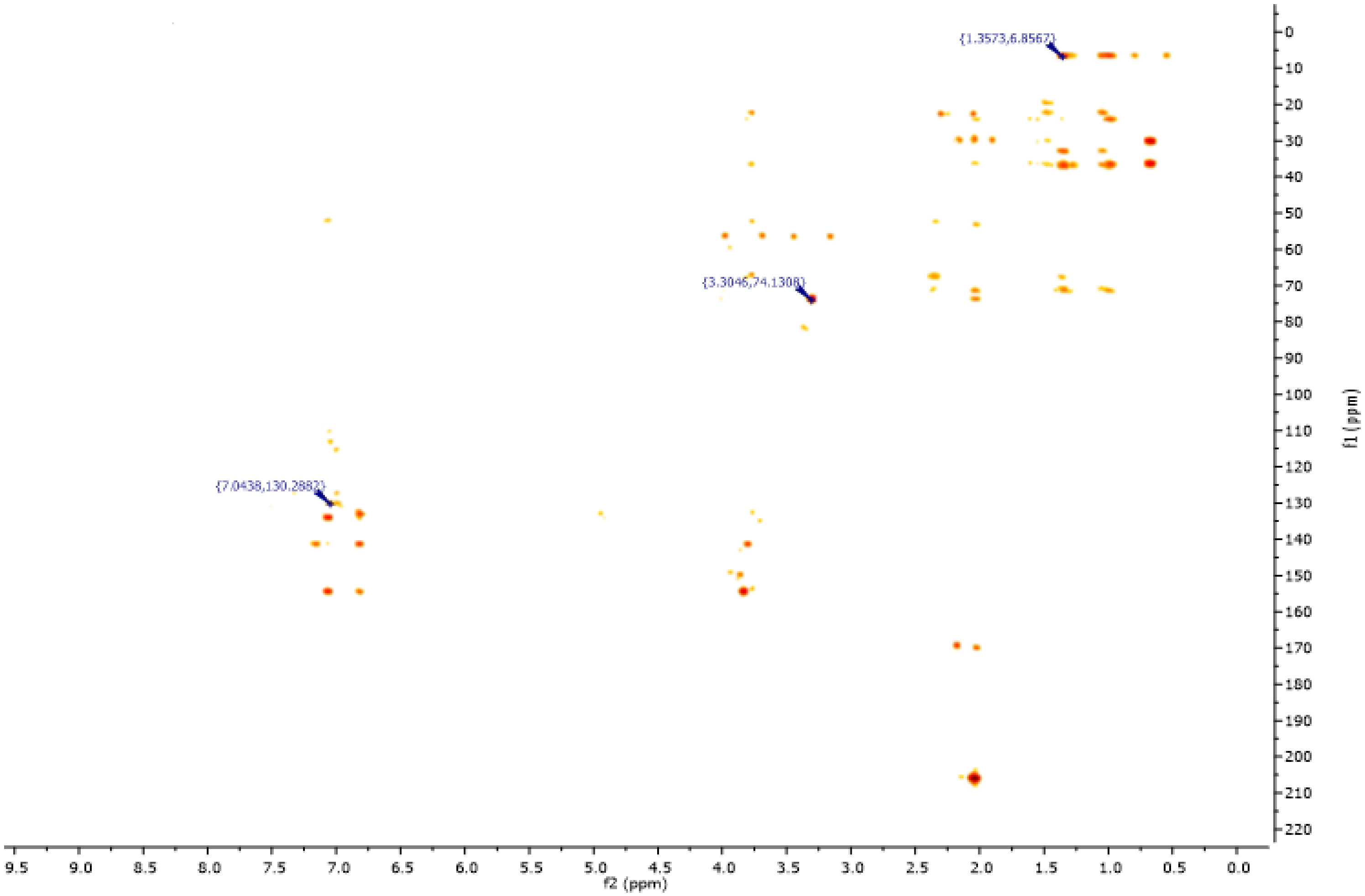

Figure S10. HMBC spectrum of **1** and **2** (acetone-d_6)

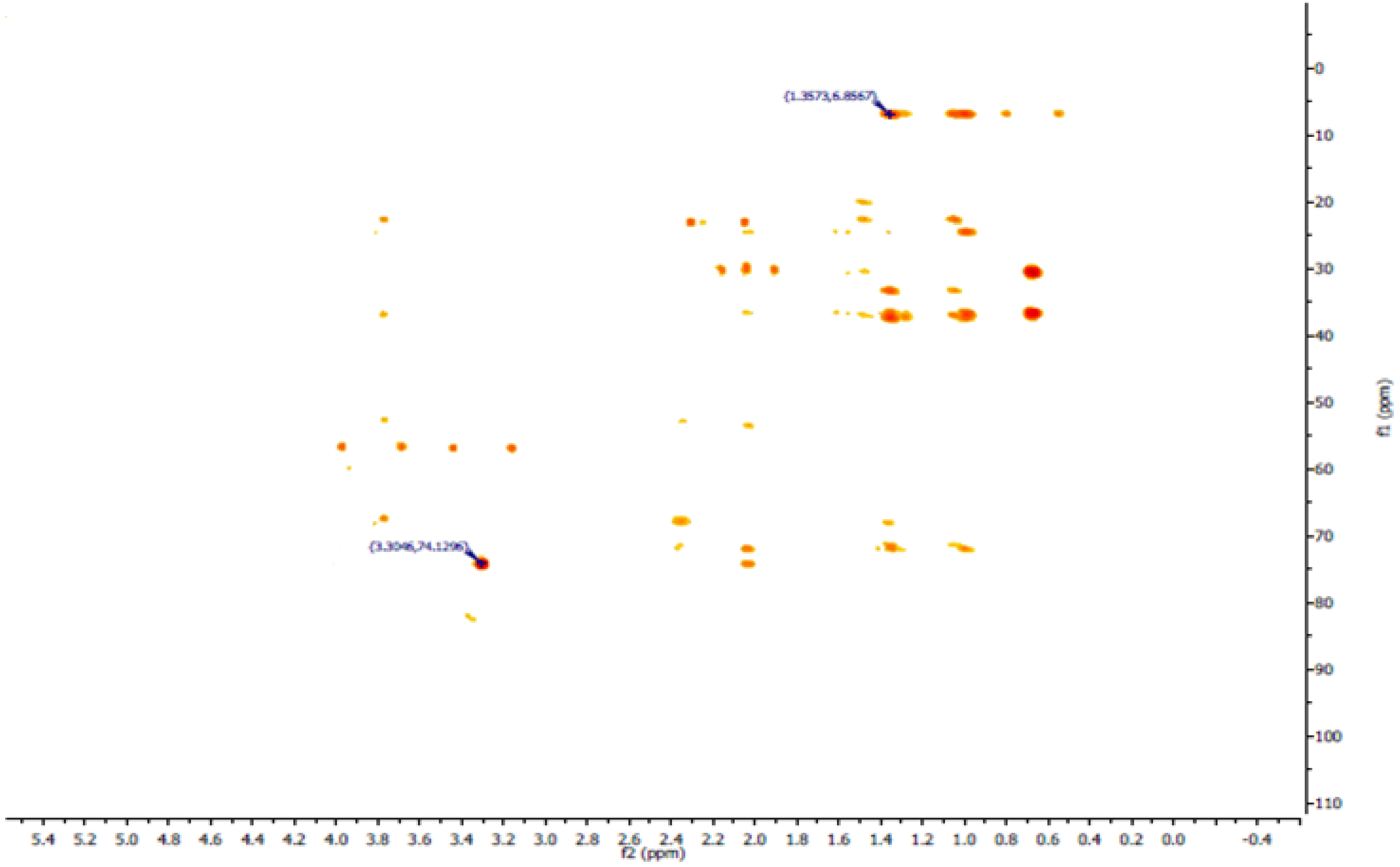

Figure S11. HMBC spectrum expansion of **1** and **2** (acetone-d_6)

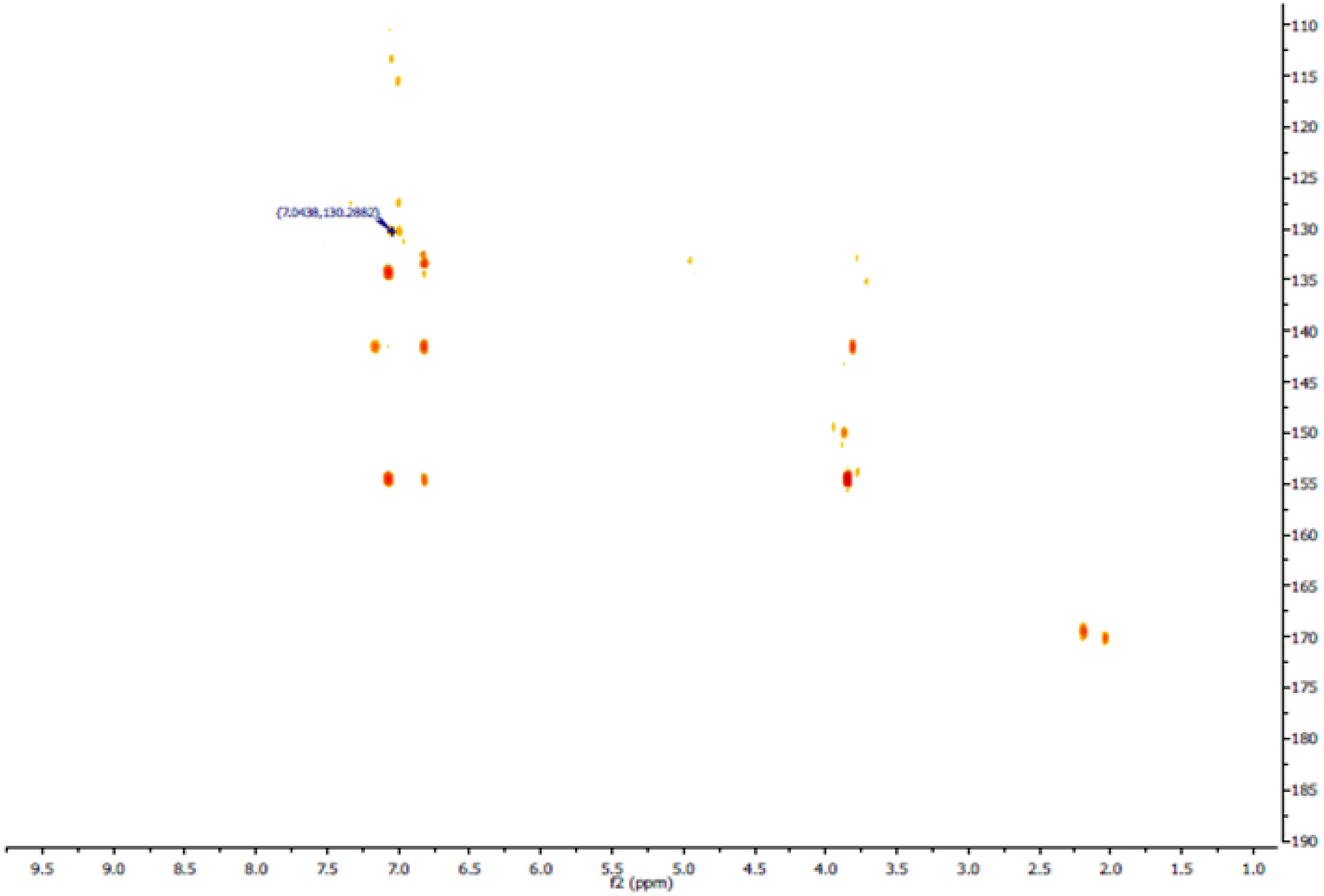

Figure S12. HMBC spectrum expansion of **1** and **2** (acetone-d_6)

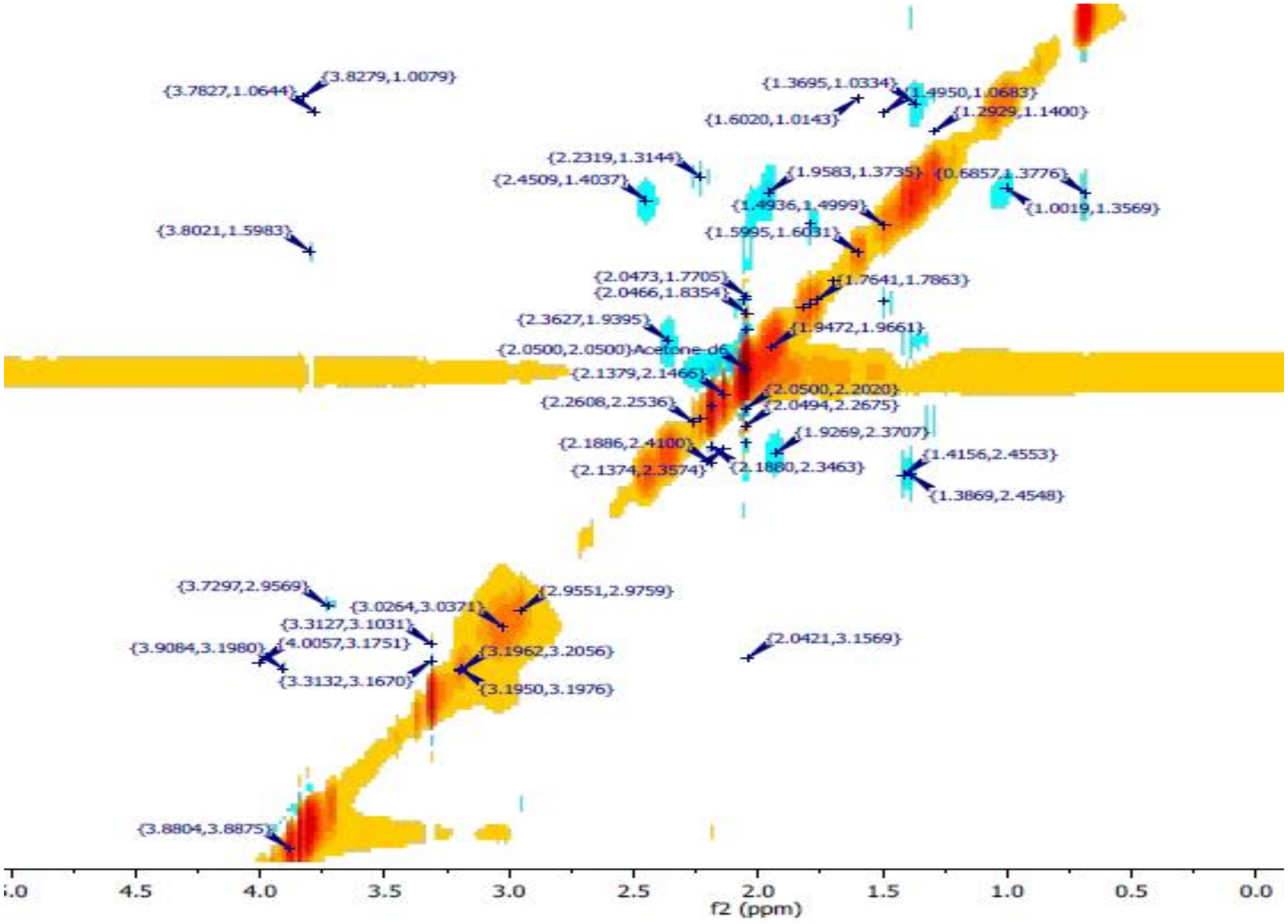

Figure S13. NOESY spectrum expansion of **1** and **2** (acetone-d_6)
